# Bioactive Lipid Compounds and Nutritional Potential of Glyceride Oils from Flower Buds and Fruits of *Lagerstroemia indica* L. Cultivar ‘Hopi’ Grown in Bulgaria

**DOI:** 10.3390/foods14091449

**Published:** 2025-04-22

**Authors:** Olga Teneva, Zhana Petkova, Maria Angelova-Romova, Ginka Antova

**Affiliations:** Department of Chemical Technology, Faculty of Chemistry, University of Plovdiv “Paisii Hilendarski”, 24 Tzar Asen Street, 4000 Plovdiv, Bulgaria; olga@uni-plovdiv.bg (O.T.); zhanapetkova@uni-plovdiv.bg (Z.P.); maioan@uni-plovdiv.bg (M.A.-R.)

**Keywords:** *Lagerstroemia indica* L., flower buds, fruit, composition, glyceride oil, biologically active components

## Abstract

This study explored the bioactive lipid compounds and assessed the nutritional potential of glyceride oils extracted from flower buds and fruits of *Lagerstroemia indica* L. cultivar ‘Hopi’ grown in Bulgaria. The study focused on chemical composition, fatty acid composition, and the content of biologically active compounds of the oils. The results indicated relatively higher levels of glyceride oil in fruits (F), 14.8%, compared to flower buds (FB), 3.3%. A similar trend was observed for protein content—15.7% (F) vs. 8.7% (FB). Total sterol and phospholipid content was higher in the FB than in the F. The primary components of sterol composition were β-sitosterol and campesterol. The main individual phospholipid classes were phosphatidylinositol and phosphatidylcholine in both oils. Linoleic acid was the predominant component (77.3% in the oil from the FB vs. 86.0% in the oil from the F), followed by comparable quantities of oleic acid. Palmitic acid was the main saturated fatty acid. To evaluate the therapeutic effect of isolated glyceride oils, the following indices were measured: index of atherogenicity, thrombogenicity, and hypocholesterolemic/hypercholesterolemic ratio. The study sought to compare the levels of glyceride oil, protein content, total sterol and phospholipid content, and to identify the main components of fatty acids, sterols, and phospholipids in the flower buds and fruits and their oils of *L. indica* cultivar ‘Hopi’.

## 1. Introduction

*Lagerstroemia indica* L., commonly known as a crape myrtle (family Lythraceae, genus *Lagerstroemia*), originates from Asia and is now widespread in tropical regions. It is cultivated in many Chinese provinces, including Guangdong, Shandong, Jiangsu, Yunnan, Hubei, Henan, Guizhou, and Hebei [1]. This shrub can reach a height of up to 3 m [2]. The flowers can be reddish, purple, or white, with the white variety known as *L. indica* Linn. f. alba (Nichols.) [3]. Members of this family are cold resistant and can thrive not only in China but also in South Korea, Japan, Taiwan, and India [4,5,6,7].

The plant *L. indica* known as ‘*Gol Turi*’ is a medicinal herb with good pharmacological effects such as antibacterial, antioxidant, analgesic, etc. [3,8]. There is also information about the perfumery and cosmetic uses of aromatic substances contained in *L. indica* L.

All plant parts—leaves, bark, flowers, and fruits—are used medicinally, exhibiting laxative effects [1]. The plant has been employed in managing blood pressure, urinary function, kidney purification, diarrhea treatment, pain relief, cholesterol control, bowel movement support, and diabetes management due to the presence of hypoglycemic compounds, comparable to 6–7.7 units of insulin [9]. Essential oils from the leaves and flowers of *L. indica* are widely used across pharmaceutical, food, and retail industries [7]. Additionally, the plant demonstrates early seed production, high yield, broad ecological adaptability, and an extended productive period [10]. The chemical composition of *L. indica* includes flavonoids, coumarins, phenolic compounds, alkaloids, and terpenoids [11,12,13]. The seeds are recognized for their sedative alkaloids, while the bark and shells function as stimulants and antipyretics [14]. The leaves and flowers serve as cleansing agents and detoxifiers that neutralize toxic chemicals. Nutritionally, *L. indica* leaves contain 22.53% protein, 37.25% carbohydrates, and 12.23% ash on a dry-weight basis, with mineral components such as potassium, calcium, magnesium, phosphorus, sodium, and sulfur [15].

Data on the fatty acid composition of glyceride oil from members of the Lythraceae family remain limited. In cold-pressed seed oil from three variety groups—*L. indica* Amabilis, *L. indica* Rubra, and *L. indica* Alba—seventeen identical fatty acids were identified at varying levels, with linoleic acid predominating (70.91–72.87%) and oleic acid ranging from 11.52 to 13.74% [10].

Compared to conventional vegetable oils, *L. indica* oil has distinct characteristics. Sunflower oil typically contains 60–65% linoleic acid [16], whereas *L. indica* oil has a higher content. Canola oil is known for its balanced oleic acid level [17], a trait similarly found in *L. indica* oil. Regarding sterols, soybean oil contains β-sitosterol, associated with LDL cholesterol reduction [18], and *L. indica* oil also contains phytosterols in significant amounts. Phospholipids—limited in most refined oils—are present in *L. indica* oil and support cell signaling and neurological health [19]. The indices of atherogenicity (IA) and thrombogenicity (IT) suggest *L. indica* oil has values similar to olive oil, potentially lowering the risk of atherosclerosis and thrombosis [20]. The hypocholesterolemic/hypercholesterolemic (HH) ratio suggests that oils with values above 2.5 may offer cholesterol-lowering benefits indicating a favorable profile for *L. indica* oil.

Based on the literature review, it was found that there is no information available on the chemical composition of the flower buds and fruits of *L. indica* L., as well as the glyceride oils derived from them. No studies have reported on the fatty acid composition of seed oil from the exact cultivar ‘Hopi’ of *L. indica*. Additionally, there is a lack of information regarding the presence of lipid-soluble bioactive compounds, such as sterols, tocopherols, and phospholipids, in the flower buds and fruits. Consequently, the aim of this study was to comprehensively characterize the chemical composition (total proteins, lipids, carbohydrates, fibers, ash) and the glyceride oils from the flower buds and fruits of *L. indica* L. cultivar ‘Hopi’ grown in Bulgaria, a plant source that has not been previously explored in detail. This study provides new data on the composition and nutritional potential of these glyceride oils. The characterization of fatty acids, sterols, tocopherols, and phospholipids, along with health-related lipid indices, highlights the potential usefulness of these oils in food and nutrition-related applications. The results may contribute to the broader understanding of plant-based oils and their possible roles in developing functional food ingredients or supplements, particularly in the context of promoting balanced and health-supportive diets.

## 2. Materials and Methods

### 2.1. Samples

Flower buds and fully ripe fruits of *L. indica* L. cultivar ‘Hopi’ were harvested from 30 plants in Plovdiv, Bulgaria. The collected samples were pooled, and representative samples were taken for analysis. The flowering time was from June to September and fruiting time was from July to October. The flower buds were collected in September 2024, while the fruits were collected in October 2024. The initial moisture content of the FB was approximately 65.0%. Both, FB and F were air dried to moisture contents of 9.7% and 8.3%, respectively. After achieving these moisture levels, all analyses were conducted.

### 2.2. Chemical Composition

Soxhlet extraction with n-hexane (CAS No. 110-54-3, Merck, Darmstadt, Germany) was used for isolation of the lipids [21]. The protein content was determined using a Kjeldahl apparatus (Velp Scientifica Srl, Usmate, Italy) after the mineralization of the sample and the distillation of the solution in UDK 127 [22]. Total carbohydrates were calculated as follows: 100 − (weight in grams [protein + lipids + moisture + ash] in 100 g of dry seeds) [23]. The content of fibers, ash, and moisture were determined according to AOAC [22]. The energy value (EV) in kcal/100 g was calculated as follows:

EV (kcal/100 g) = % proteins × 4.0 + % carbohydrates × 4.0 + % lipids × 9.0.

### 2.3. Lipid Composition

#### 2.3.1. Fatty Acid Composition

The fatty acid composition was determined by gas chromatograph (GC) after trans-esterification of the oils [24,25]. The GC was Agilent 8860 (Santa Clara, CA, USA) with a flame ionization detector (FID) and a capillary column DB-Fast FAME (Agilent, USA): 30 m × 0.25 mm × 0.25 μm. The conditions were column temperature—70 °C (1 min), increasing at a rate of 5 °C/min to 250 °C (hold 3 min); injector temperature—270 °C; detector temperature—300 °C. For identification, a standard mixture of FAMEs was used (37 comp. FAME mix, Supelco, Bellefonte, PA, USA) and subjected to GC analysis.

#### 2.3.2. Index of Atherogenicity (IA), Index of Thrombogenicity (IT), Hypocholesterolemic/Hypercholesterolemic Ratio (HH)

The following formulae were used to calculate the IA, IT and HH [20,26]:

IA = (C12:0 + 4 × C14:0 + C16:0)/(∑MUFA + ∑PUFA)

IT = (C14:0 + C16:0 + C18:0)/[(0.5 × ∑MUFA) + (0.5 × ∑n-6 PUFA) + (3 × ∑n-3 PUFA) + (∑n-3 PUFA/∑n-6 PUFA)]

HH ratio = [C18:1 (n-9) + C18:2 (n-6) + C18:3 (n-6) + C18:3 (n-3) + C20:2 (n-6) + C20:3 (n-6) + C20:4 (n-6)] + C22:6 (n-3)/(C12:0 + C14:0 + C16:0)

where C12:0—lauric acid, C14:0—myristic acid, C16:0—palmitic acid, C18:0—stearic acid, C18:1 (n-9)—oleic acid, C18:2 (n-6)—linoleic acid, C18:3 (n-3)—α-linolenic acid, C18:3 (n-6)—γ-linolenic acid, C20:2 (n-6)—eicosadienoic acid, C20:3 (n-6)—dihomo-γ-linolenic acid, C20:4 (n-6)—arachidonic acid, C22:6 (n-3)—docosahexaenoic acid; ∑MUFA is the amount of the monounsaturated fatty acids, ∑PUFA—polyunsaturated fatty acids, ∑n-6 PUFA—polyunsaturated fatty acids (n-6), ∑n-3 PUFA—polyunsaturated fatty acids (n-3).

#### 2.3.3. Sterols

The oil was saponified and the unsaponifiable matter was extracted [27]. The sterols were isolated by thin-layer chromatography (TLC) and were determined spectrophotometrically [28]. The individual sterol composition was analyzed on Agilent 8860 (Santa Clara, CA, USA) with a DB 5 capillary column (25 m × 0.25 mm × 0.25 µm). The conditions were as follows: starting from 90 °C (3 min) to 290 °C at a rate of 15 °C/min, an increase at a rate of 4 °C/min to 310 °C (10 min); detector temperature—320 °C; injector temperature—300 °C. For identification, a standard mixture of sterols was used (75% β-sitosterol and 10% campesterol, Acros Organics, Morris Plains, NJ, USA; cholesterol, Acros Organics, Morris Plains, NJ, USA; stigmasterol, Sigma-Aldrich, St. Louis, MO, USA) [29].

#### 2.3.4. Tocopherols

The total content of tocopherols and individual tocopherols were determined using high-performance liquid chromatography (HPLC) on a Merck-Hitachi system (Burladingen, Germany) with a fluorescence detector F-1050 (Merck-Hitachi, Burladingen, Germany) and column Nucleosil Si 50-5 (250 mm × 4 mm). The mobile phase was n-hexane/dioxane, 96:4 (*v*/*v*) (n-hexane, CAS No. 110-54-3 and 1,4-dioxane, CAS No. 123-91-1; Merck, Darmstadt, Germany) [30].

#### 2.3.5. Phospholipids

The individual classes of phospholipids were isolated using two-dimensional TLC and their content was determined spectrophotometrically [31,32].

### 2.4. Refractive Index and Iodine Value

The refractive index was measured by Abbe refractometer.

Iodine value (IV) was determined on the basis of the fatty acid composition using the following formula:

IV = [(90 × % C18:1) + (181 × % C18:2) + (274 × % C18:3)]/100, gI_2_/100 g

### 2.5. Statistical Analysis

The results are expressed as the mean values of three parallel determinations ± standard deviations. The analyses were performed in triplicate (*n* = 3). Data were analyzed using one-way ANOVA followed by Duncan’s test for multiple comparisons (*p* < 0.05).

## 3. Results and Discussion

The chemical composition of flower buds and fruits of *L. indica* cultivar ‘Hopi’ was determined. The results are presented in Table 1.

The results indicated a high level of glyceride oil in the fruits (F) at 14.8%, compared to the flower buds (FB) at 3.3%. The data for F correlated with the oil content of cold-pressed crude oils for three variety groups of *L. indica* reported by Jianmin et al. [10] as follows: 17.78% for Amabilis, 16.93% for Rubra, and 16.86% for Alba. The other species of family Lythraceae, genus *Lagerstroemia*—namely *L. speciosa*—is characterized by 16.048% glyceride oil content [33]. For flower buds, physiological changes during development impact oil content. The accumulation of glyceride oils in fruits and flower buds involves intricate metabolic pathways. In fruits, lipid biosynthesis is controlled by transcriptional networks that orchestrate the assembly of fatty acids and triacylglycerols [34]. The protein content was 15.7% in F, while FB had half that content at 8.7%. In flower buds, energy is primarily allocated toward reproductive development rather than protein storage. The lower protein content in flower buds is associated with hormonal regulation, which governs their growth and maturation [35]. These results are lower than those reported by Niranjan and Sudarshana [15] for other parts of *L. indica* Linn., specifically the leaves, which had a protein content of 22.53%. Protein accumulation in fruits is shaped by nitrogen metabolism, amino acid biosynthesis, and storage protein formation. Throughout fruit development, key enzymes involved in nitrogen assimilation—such as nitrate reductase and glutamine synthetase—play a vital role in protein synthesis [36].

The carbohydrate content was significantly higher in FB than in F (72.0% vs. 53.1%), exceeding the levels reported by Niranjan and Sudarshana [15] for the leaves (37.25%). The higher carbohydrate content in flower buds may be due to the high metabolic activity during floral organogenesis, where sugars not only fuel growth but also act as developmental signals [37]. The ideal ratio of carbohydrates to proteins in edible plants depends on dietary needs. A balanced diet typically includes a range of foods with varying carbohydrate-to-protein ratios. For general dietary balance, an average ratio between 4:1 and 7:1 may be suitable, although individual nutritional needs can influence this [38]. The studies demonstrated a high and comparable quantity of crude fibers among the samples tested (31.7% in FB vs. 28.3% in F). A prior study demonstrated that the intake of soluble dietary fiber significantly contributes to the alleviation of cardiovascular disease and diabetes, reduction of inflammation and cholesterol levels, and regulation of gut microbiota [39].

The ash content, which refers to the inorganic residue remaining after the organic matter has been burned away, was determined. The results showed that the ash level of the analyzed samples was as follows: 8.2% in F vs. 6.3% in FB. Our results were lower than those reported by Niranjan and Sudarshana [15] for the leaves of *L. indica* Linn., which was 12.23%. The energy value of FB and F was calculated (408.2 vs. 352.9 kcal/100 g) and was found to be similar to that of herbs or medicinal plants (314 kcal/100 g) [40]. The differences in these indicators may be due to the agro-meteorological conditions in which the plants grew, as well as the fact that different morphological parts of the plants were studied.

Overall, the observed differences between the chemical composition of the flower buds and fruits can be attributed to their distinct physiological roles and metabolic demands. The fruits probably prioritize energy storage through elevated glyceride oil and protein levels.

The biologically active components can be found in foods and natural products. They can affect the body and its functions such as improving immunity, reducing inflammation, or protecting against diseases [41]. The analyzed biologically active compounds in the oils of flower buds and fruits were unsaponifiable matter, sterols, phospholipids, and tocopherols. The results are given in Table 2. All of these components are often studied for their potential health benefits and preventing chronic diseases.

The results indicated that the unsaponifiable matter content in fruits (F) was approximately four times lower than in flower buds (FB), with values of 4.6% and 19.6%, respectively. The results for the fruits are about twice as high as those reported by Basu et al. [33] for *L. speciosa* seed oil (1.9%). Flower buds are in an early developmental stage, where they accumulate various protective and growth-promoting substances. These substances are often unsaponifiable, contributing to the higher content in buds compared to mature fruits. This is probably one of the primary reasons for the elevated unsaponifiable matter content in FB.

The total sterol content was determined, revealing that the content in FB is around three times higher than in F, with values of 4.1% and 1.6%, respectively. The higher sterol content in flower buds can be linked to elevated membrane synthesis demands during organ formation [42]. Sterols are essential components of cell membranes and play a crucial role in plant growth and development. The reason for this is probably that during the early stages of development, such as in flower buds, plants may have higher sterol biosynthesis activity to support rapid cell division and growth. Higher sterol content in flower buds may provide additional protection by strengthening cell membranes and enhancing the plant’s defense mechanisms. Plants allocate resources differently at various stages of development. In the case of flower buds, the plant may prioritize the production of sterols to ensure successful reproduction. Once the flowers develop into fruits, the metabolic focus may shift towards other compounds, such as sugars and organic acids, which are essential for fruit development and maturation [43].

The individual sterol composition was also determined. The flower buds and fruits have almost equal individual sterol composition. β-Sitosterol serves both structural and hormonal functions [42]. It was predominant in both samples followed by campesterol. The quantities of all sterols were comparable, except for two components: 24-methylenecholesterol and stigmasterol. The content of 24-methylenecholesterol was approximately five times higher in FB than in fruits F (10.3% vs. 2.4%), which was likely due to the lower content of stigmasterol in FB compared to F (2.8% vs. 13.4%). 24-Methylenecholesterol is a type of sterol found in various plant tissues, including flower buds and fruits. It helps in the development and growth of the buds. According to Lusby et al. [44], the presence of 24-Methylenecholesterol in flower buds can be linked to various physiological processes, including cell division and differentiation. In fruits, it is also present but in varying quantities compared to flower buds. The concentration of this sterol can differ depending on the type of fruit and its developmental stage. In some cases, the content of 24-methylenecholesterol in fruits may be lower than in flower buds, as observed in certain studies [45]. The content of cholesterol for Δ^7^, campesterol, and Δ^7^, avenasterol, was identified below 0.5% in both samples.

Tocopherols, recognized as the most important natural antioxidants, were identified in the glyceride oils of flower buds and fruits from *L. indica*. The tocopherol content was analyzed. The results showed higher amounts of total tocopherols in the FB compared to F (80.0 vs. 50.6 mg/kg). Only α-tocopherol was identified in the flower bud and only γ-tocopherol in F.

**Figure 1:**
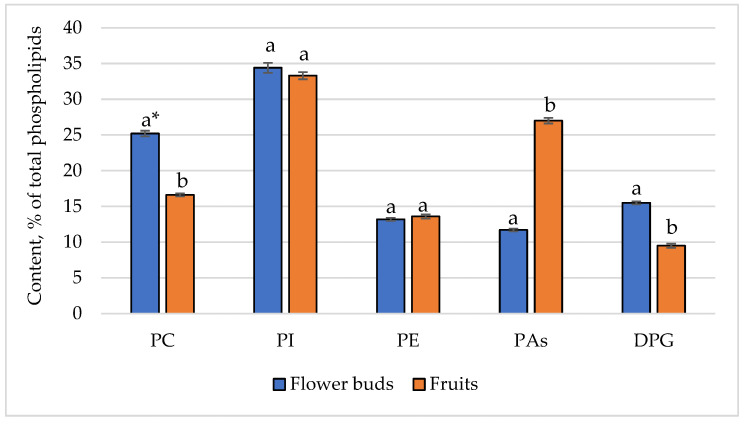
Phospholipid composition of glyceride oils of flower buds and fruits from *L. Indica* cultivar ‘Hopi’. * The samples were analyzed in triplicate (*n* = 3) and the results are expressed as mean ± standard deviation. Different small letters in a row depict significant differences in the results (*p* < 0.05); PC—phosphatidylcholine; PI—phosphatidylinositol; PE—phosphatidylethanolamine; PAs—phosphatidic acids; DPG—diphosphatidylglycerol.

Phospholipids are involved in various metabolic processes, including the synthesis of other lipids. They are integral to the formation of new membranes during cell division. This is particularly important during the rapid growth phases of flower buds and fruits. This likely explains the higher phospholipid content observed in flower buds, which is 2.8%, compared to 0.8% in fruits. The individual phospholipid composition was also analyzed, and the results are present in Figure 1.

The identified individual phospholipids included phosphatidylcholine, phosphatidylinositol, phosphatidylethanolamine, phosphatidic acids, and diphosphatidylglycerol. In FB and F, the primary components were phosphatidylinositol (34.4% vs. 33.3%), phosphatidylcholine (25.2% vs. 16.6%), and phosphatidic acids (11.7% vs. 27.0%). The phosphatidylethanolamine content was similar in FB and F (13.2% vs. 13.6%), likely due to its vital role as a key phospholipid in essential cellular processes, including membrane fusion, signaling, and lipid metabolism, which are critical for the development of both flower buds and fruits. Some differences were observed in the levels of the remaining analyzed phospholipids. The higher content of phosphatidylcholine in the FB may be due to the increased need for cell membrane synthesis and stability during their rapid growth and development [46]. On the other hand, flower buds are highly metabolically active tissues, requiring efficient cellular processes for growth and differentiation. Phosphatidylcholine plays a crucial role in membrane fluidity and function, supporting these metabolic activities. The higher content of phosphatidic acids in fruits compared to flower buds is likely due to the increased need for membrane biosynthesis, signal transduction, stress response, and metabolic activity during fruit development and ripening [47]. A high content of diphosphatidylglycerol was also identified in flower buds (15.5% vs. 9.5%). The reason for this is likely similar to that for phosphatidylcholine.

The fatty acid composition, some physicochemical characteristics, and the main lipid indices of the oils are presented in Table 3.

A total of twenty-four fatty acids were identified, with three being predominant. Linoleic acid was the main component (86.0% vs. 77.3%, F vs. FB), followed by oleic acid in similar quantities (approximately 6.0%). Palmitic acid was the primary fatty acid among the saturated fatty acids, with a higher content in FB compared to F (8.1% vs. 5.8%). Linoleic acid, being an essential fatty acid, is crucial for the growth and development of seeds. The higher content of linoleic acid in fruits compared to flower buds can be attributed to the different roles and metabolic processes in these plant parts. Fruits often serve as storage organs for energy and nutrients while the flower buds are primarily involved in reproduction and may not require as much energy storage in the form of fatty acids.

The results regarding fatty acid composition do not differ significantly from those reported by Jianmin et al. [10] for *L. indica* seed oils, as shown in Table 3. The fatty acid composition of the Amabilis group was found to be the closest to that of flower buds, except for the content of oleic acid, which was lower in both FB and F oils. On the other hand, Basu et al. [33] reported data for *L. speciosa* seed oil showing a higher content of palmitic acid (14.46%) and stearic acid (7.74%), and a lower quantity of linoleic acid (48.57%) compared to our results. The data for the total content of saturated (SFA) and unsaturated fatty (UFA) acids are comparable with the literature. Only the SFA content in the fruits was significantly lower than that in the other samples [10].

Iodine values are frequently utilized to measure the level of unsaturation in fatty acids. This unsaturation exists in the form of double bonds, which interact with iodine compounds. The greater the iodine number, the higher the quantity of C=C bonds in the oils. The iodine value was calculated and found to be over 140 gI_2_/100 g for the examined samples which classifies the analyzed oils as drying oils [48]. Our results are a little bit higher than reported by Basu et al. [33] for *L. speciosa* seed oil where it was found to be 121.93 gI_2_/100 g. The refractive index, which indicates the purity and quality of the oil, was almost the same for both oils (1.4802 for FB vs. 1.4825 for F). These values were higher than reported in CodexStan-210 [49] for the traditional edible oils as sunflower oil (1.461) and sesame oil (1.465)

Various significant lipid indices can be derived from the identified individual fatty acid composition. These indices suggest potential health benefits of the glyceride oils, including anti-atherogenic and anti-thrombogenic properties. The indices of atherogenicity and thrombogenicity reflect the health benefits of the lipids. A higher IA indicates a greater risk of atherosclerosis and related cardiovascular diseases. The IA is particularly useful in evaluating the balance between harmful and beneficial cholesterol levels in the blood [50]. A higher IT indicates a greater risk of thrombosis, which can lead to conditions such as heart attacks and strokes [51] and a HH ratio lower than 1 indicates a greater potential for raising blood cholesterol levels, which can increase the risk of cardiovascular diseases [52].

Lower values indicate stronger anti-atherogenic and anti-thrombogenic effects of the oils. The numbers of these indices of the *L. indica* cultivar ‘Hopi’ oil from flower buds and fruits were low (0.1021 vs. 0.0628, IA and 1.1008 vs. 0.9928, IT). That means good beneficial effects.

An HH ratio of approximately 10–15 in a glyceride oil indicates a predominance of fatty acids that possess hypocholesterolemic properties over those with hypercholesterolemic ones. This suggests that the oil has a favorable fatty acid profile, potentially expressing beneficial effects on lipid metabolism and cardiovascular health by reducing low-density lipoprotein (LDL) cholesterol levels and increasing high-density lipoprotein (HDL) cholesterol levels [53].

## 4. Conclusions

For the first time, the scientific literature presents data on the chemical and lipid composition of the flower buds and fruits of the *L. indica* cultivar ‘Hopi’, providing new insights into their nutritional and health-promoting properties. The analysis revealed that both flower buds and fruits are rich in proteins, carbohydrates, and dietary fibers, which are essential for maintaining a balanced and healthy diet. Additionally, the glyceride oils extracted from these plant parts were found to contain several lipid-soluble biologically active components, including essential fatty acids, tocopherols, sterols, and phospholipids. These bioactive compounds play crucial roles in promoting health and preventing various diseases. The presence of these biologically active components in significant amounts suggests that the glyceride oils from *L. indica* cultivar ‘Hopi’ may have potential nutritional value and could be considered as potential health oils, paving the way for further research and potential applications in the food and nutraceutical industries.

## Figures and Tables

**Table 1:** Chemical composition of flower buds and fruits of *L. indica* cultivar ‘Hopi’.

Compounds	Flower Buds	Fruits
Glyceride oil, %	3.3 ± 0.2 ^a^*	14.8 ± 0.4 ^b^
Protein, %	8.7 ± 0.2 ^a^	15.7 ± 0.2 ^b^
Carbohydrates, %	72.0 ± 0.5 ^a^	53.1 ± 0.6 ^b^
Crude fiber, %	31.6 ± 0.8 ^a^	28.3 ± 0.8 ^b^
Ash, %	6.3 ± 0.1 ^a^	8.2 ± 0.1 ^b^
Energy value, kcal/100 g	352.9 ^a^	408.2 ^b^

* The samples were analyzed in triplicate (*n* = 3) and the results are expressed as mean ± standard deviation. Different small letters in a row depict significant differences in the results (*p* < 0.05).

**Table 2:** Content of biologically active components of oils from flower buds and fruits of *L. indica* cultivar ‘Hopi’.

Biologically Active Components	Flower Buds	Fruits
Unsaponifiable matter, %	19.6 ± 0.2 ^a^*	4.6 ± 0.1 ^b^
Total sterols, %	4.1 ± 0.1 ^a^	1.6 ± 0.1 ^b^
Cholesterol, % (of total sterols)	0.4 ± 0.0 ^a^*	0.4 ± 0.1 ^a^
Campesterol, % (of total sterols)	9.3 ± 0.2 ^a^	9.2 ± 0.1 ^a^
24-Methylenecholesterol, % (of total sterols)	10.3 ± 0.2 ^a^	2.4 ± 0.1 ^b^
Stigmasterol, % (of total sterols)	2.8 ± 0.1 ^a^	13.4 ± 0.4 ^b^
β—Sitosterol, % (of total sterols)	76.5 ± 0.5 ^a^	74.3 ± 0.3 ^b^
Δ^7^—Campesterol, % (of total sterols)	0.5 ± 0.0 ^a^	0.3 ± 0.0 ^b^
Δ^7^—Avenasterol, % (of total sterols)	0.2 ± 0.0	nd **
α—Tocopherol, mg/kg	80 ± 4 ^a^	nd
γ—Tocopherol, mg/kg	nd	56 ± 2 ^a^
Phospholipids, %	2.8 ± 0.1 ^a^	0.8 ± 0.0 ^b^

* The samples were analyzed in triplicate (*n* = 3) and the results are expressed as mean ± standard deviation. Different small letters in a row depict significant differences in the results (*p* < 0.05). ** nd—not detected

**Table 3:** Fatty acid composition of glyceride oils, physicochemical characteristics, and lipid indices of flower buds and fruits from *L. indica* cultivar ‘Hopi’.

Fatty Acids (FAs) *, %		*L. indica* Cultivar ‘Hopi’		*L. indica* Amabilis [10]	*L. indica* Rubra [10]	*L. indica* Alba [10]
		Flower Buds	Fruits	Seeds	Seeds	Seeds
C8:0	Caprylic	0.3 ± 0.0 ^a^*	0.1 ± 0.0 ^b^			
C10:0	Capric	0.4 ± 0.0 ^a^	0.1 ± 0.0 ^b^			
C11:0	Undecanoic	0.1 ± 0.0	nd **			
C14:0	Myristic	0.2 ± 0.0	nd			
C15:0	Pentadecanoic	1.2 ± 0.1 ^a^	0.1 ± 0.0 ^b^			
C15:1	Pentadecenoic	0.3 ± 0.0	nd			
C16:0	Palmitic	8.1 ± 0.2 ^a^	5.8 ± 0.1 ^b^	8.94	9.54	9.60
C16:1	Palmitoleic	0.2 ± 0.0	nd			
C17:0	Margaric	0.4 ± 0.0 ^a^	0.1 ± 0.0 ^b^			
C17:1	Heptadecenoic	0.5 ± 0.0	nd			
C18:0	Stearic	1.2 ± 0.0 ^a^	1.1 ± 0.1 ^a^	2.31	2.96	2.76
C18:1 trans	Elaidic	0.7 ± 0.0	nd			
C18:1	Oleic	6.1 ± 0.1 ^a^	5.6 ± 0.1 ^b^	13.74	12.76	11.52
C18:2 trans	trans-Linoleic	0.8 ± 0.0	nd			
C18:2 (n-6)	Linoleic	77.3 ± 0.2 ^a^	86.0 ± 0.3 ^b^	71.62	70.91	72.87
C18:3 (n-6)	γ-Linolenic	0.1 ± 0.0	nd			
C18:3 (n-3)	α-Linolenic	0.6 ± 0.0 ^a^	0.3 ± 0.0 ^b^	0.55	0.69	0.49
C20:0	Arachidic	0.5 ± 0.0 ^a^	0.2 ± 0.0 ^b^			
C20:1	Gadoleic	0.2 ± 0.0 ^a^	0.3 ± 0.0 ^b^			
C20:2 (n-6)	Eicosadienoic	0.1 ± 0.0 ^a^	0.1 ± 0.0 ^a^			
C20:3 (n-6)	Dihomo-γ-linolenic acid	0.1 ± 0.0	nd	0.12	0.10	0.10
C22:0	Behenic	0.4 ± 0.0 ^a^	0.1 ±0.0 ^b^			
C22:1	Erucic	0.1 ± 0.0 ^a^	0.1 ±0.0 ^a^			
C22:6 (n-3)	Docosahexaenoic	0.1 ± 0.0	nd			
Saturated FAs		12.8	7.6	13.05	14.38	14.00
Unsaturated FAs		87.2	92.4	86.57	85.21	85.57
Monounsaturated FAs		8.1	6.0			
Polyunsaturated FAs		79.1	86.4			
**Physicochemical characteristics**						
Iodine value, g I_2_/100 g		147 ± 1 ^a^	162 ± 3 ^b^			
Refractive index		1.4802 ± 0.0002 ^a^	1.4825 ± 0.0009 ^b^			
**Lipid indices**						
Index of atherogenicity (IA)		0.1021 ± 0.0013	0.0628 ± 0.0008			
Index of thrombogenicity (IT)		1.1008 ± 0.0021	0.9928 ± 0.0014			
Hypocholesterolemic/hypercholesterolemic ratio (HH)		10.1566 ± 0.0053	15.8621 ± 0.0061			

* The samples were analyzed in triplicate (*n* = 3) and the results are expressed as mean ± standard deviation. Different small letters in a row depict significant differences in the results (*p* < 0.05); ** nd—not detected.

## Data Availability

The original contributions presented in the study are included in the article, further inquiries can be directed to the corresponding author.

## References

[1] Editorial Committee of Chinese Botany, Chinese Academy of Sciences (1983). Flora Reipublicae Popularis Sinicae.

[2] Ghahreman A. (1993). Chromite Iran.

[3] Chang M., Ahmed A.F., Cui L. (2023). The hypoglycemic effect of *Lagerstroemia indica* L. and *Lagerstroemia indica* L. f. alba (Nichols.) Rehd. in vitro and in vivo. J. Future Foods.

[4] Yoziev L.K. (1996). Introduction of *Lagerstroemia indica* in the conditions of Southern Uzbekistan. Introduction and Acclimatization of Plants.

[5] Muratgeldiev N. (1967). *Lagerstroemia indica* in Ashgabat. Bull. Head. Nerd. Sada.

[6] Kuznetsova V.M. (1987). Collection of *Lagerstroemia indica* in the Nikitsky Botanical Garden. Bull. State Nikit. Bot. Gard..

[7] Sharopova M.A. (2005). Characteristics of growth and development of *Lagerstroemia* and *Cesalpinia* in different phases of ontogenesis. J. Biol. Uzbekistan.

[8] Noori Naeiji M., Vahdat S.M. (2024). A study on the antioxidant and antimicrobial properties of the aerial parts extract of *Lagerstroemia indica* L.. J. Med. Plants By-Prod..

[9] Sharopova M.A., Uzakov Z.Z., Xaitov I.Y. (2024). Preliminary results on the chemical components of *Lagerstroemia indica* L. in the conditions of southern Uzbekistan. E3S Web Conf..

[10] Jianmin H., Boxin H., Zhili S., Qiangfa Y. (2013). The fatty acid composition of *Lagerstroemia indica* seed oil determined by GC/MS. J. Agric..

[11] Zhang D., Ni G., Tang Y.J. (2005). Chemical constituents in stem-leaves of *Lagerstroemia indica*. Chin. Tradit. Herbal Drugs.

[12] Labib R.M., Ayoub N.A., Singab A.B., Al-Azizi M.M., Sleem A. (2013). Chemical constituents and pharmacological studies of *Lagerstroemia indica*. Phytopharmacology.

[13] Chen Y., Li S.-W., Yin F.-Z., Yang M., Huan X.-J., Miao Z.-H., Wang X.-M., Guo Y.-W. (2021). Lagerindicine, a new pyrrole alkaloid isolated from the flowers of *Lagerstroemia indica* Linnaeus. Nat. Prod. Bioprospect..

[14] Sharopova M.A. (2000). On the Ecological Characteristics of Acclimatized Lagerstroemia indica in South Uzbekistan, Tashkent.

[15] Niranjan M.H., Sudarshana M.S. (2010). Preliminary phytochemical studies of *Lagerstroemia indica* Linn. J. Pharm. Res..

[16] Gunstone F.D. (2002). Food Applications of Lipids. Food Lipids.

[17] Kris-Etherton P.M. (1999). Monounsaturated fatty acids and risk of cardiovascular disease. Circulation.

[18] Moreau R.A., Whitaker B.D., Hicks K.B. (2002). Phytosterols, phytostanols, and their conjugates in foods: Structural diversity, quantitative analysis, and health-promoting uses. Prog. Lipid Res..

[19] Van Meer G., Voelker D.R., Feigenson G.W. (2008). Membrane lipids: Where they are and how they behave. Nat. Rev. Mol. Cell Biol..

[20] Ulbricht T., Southgate D. (1991). Coronary heart disease: Seven dietary factors. Lancet.

[21] (2014). Oilseeds—Determination of Oil Content (Reference Method).

[22] AOAC—Association of Official Analytical Chemists (2016). Official Methods of Analysis.

[23] FAO (2003). Food Energy—Methods of Analysis and Conversion Factors.

[24] (2014). Animal and Vegetable Fats and Oils. Gas Chromatography of Fatty Acid Methyl Esters—Part 1: Guidelines on Modern Gas Chromatography of Fatty Acid Methyl Esters.

[25] (2017). Animal and Vegetable Fat and Oils. Gas Chromatography of Fatty Acid Methyl Esters—Part 2: Preparation of Methyl Esters of Fatty Acids.

[26] Santos-Silva J., Bessa R.J.B., Santos-Silva F. (2002). Effect of genotype, feeding system and slaughter weight on the quality of light lambs: Fatty and composition of meat. Livest. Prod. Sci..

[27] (2000). Animal and Vegetable Fats and Oils. Determination of Unsaponifiable Matter. Method Using Hexane Extraction.

[28] Ivanov S., Bitcheva P., Konova B. (1972). Méthode de détermination chromatographyque et colorimétrique des phytosterols dans les huiles végétales et les concentrates steroliques. Rev. Fr. Corps Gras.

[29] (2014). Animal and Vegetable Fats and Oils. Determination of Individual and Total Sterols Contents. Gas Chromatographic Method.

[30] (2016). Animal and Vegetable Fats and Oils. Determination of Tocopherol and Tocotrienol Contents by High-Performance Liquid Chromatography.

[31] Schneiter R., Daum G., Xiao W. (2006). Analysis of yeast lipids. Methods in Molecular Biology.

[32] (2014). Animal and Vegetable Fats and Oils. Determination of Phosphorus Content. Part 1: Colorimetric Method.

[33] Basu S., Kundu P., Sinhababu A. (2015). Characterization of fatty acid and sterol composition of seed lipid of *Lagerstroemia speciosa* Pers. Res. Chem. Intermed..

[34] Tranbarger T.J., Dussert S., Joët T., Argout X., Summo M., Champion A., Cros D., Omore A., Nouy B., Morcillo F. (2011). Regulatory Mechanisms Underlying Oil Palm Fruit Mesocarp Maturation, Ripening, and Functional Specialization in Lipid and Carotenoid Metabolism. Plant Physiol..

[35] Kumari R., Hamal U., Sharma N., Tandon R., Shivanna K., Koul M. (2020). Resource Allocation in Flowering Plants: Concept and Implications. Resource Allocation in Flowering Plants: Concept and Implications.

[36] Famiani F., Bonghi C., Chen Z.H., Drincovich M.F., Farinelli D., Lara M.V., Proietti S., Rosati A., Vizzotto G., Walker R.P. (2020). Stone Fruits: Growth and Nitrogen and Organic Acid Metabolism in the Fruits and Seeds—A Review. Front. Plant Sci..

[37] Rolland F., Baena-Gonzalez E., Sheen J. (2006). Sugar Sensing and Signaling in Plants: Conserved and Novel Mechanisms. Annu. Rev. Plant Biol..

[38] Nestorova V. (2014). Food Hygiene and Food Legislation.

[39] Tian J., Gong Y., Li J. (2022). Nutritional attributes and phenolic composition of flower and bud of *Sophora japonica* L. and *Robinia pseudoacacia* L.. Molecules.

[40] Teneva O., Petkova Z., Antova G., Angelova-Romova M., Stoyanov P., Todorov K., Mladenova T., Radoukova T., Mladenov R., Petkov V. (2024). Chemical composition and lipid bioactive components of *Centaurea thracica* dwelling in Bulgaria. Molecules.

[41] Bansal M., Poonia A., Kolluri S.R.P., Vasundhara, Thakur M., Belwal T. (2023). Introduction on Bioactive Compounds, Sources and Their Potential Applications.

[42] Schaller H. (2003). The Role of Sterols in Plant Growth and Development. Prog. Lipid Res..

[43] Grunwald C. (1978). Function of sterols. Philos. Trans. R. Soc. Lond. B Biol. Sci..

[44] Lusby W.R., Oliver J.E., McKibben G.H., Thompson M.J. (1987). Free and esterified sterols of cotton buds and anthers. Lipids.

[45] Yang J., Li C., Zhang Y. (2021). Engineering of *Saccharomyces cerevisiae* for 24-methylene-cholesterol production. Biomolecules.

[46] Zienkiewicz A., Wenk M. (2017). Phosphatidylcholine in plants: Functional diversity. Encyclopedia of Lipidomics.

[47] Ischebeck T., Wenk M. (2017). Phosphatidic acid in plants: Functional diversity. Encyclopedia of Lipidomics.

[48] Kelle H.I., Udeozo I.P. (2015). Characterization of the chemical properties of some selected refined vegetable oils commonly sold in Nigeria. Br. J. Appl. Sci. Technol..

[49] Codex Alimentarius Commission (2008). Codex-Stan 210: Codex Standard for Named Vegetable Oils.

[50] Assempoor R., Daneshvar M.S., Taghvaei A. (2025). Atherogenic index of plasma and coronary artery disease: A systematic review and meta-analysis of observational studies. Cardiovasc. Diabetol..

[51] Fehily A.M., Pickering J.E., Yarnell J.W.G. (1994). Dietary indices of atherogenicity and thrombogenicity and ischaemic heart disease risk: The Caerphilly Prospective Study. Br. J. Nutr..

[52] Bhatnagar D., Soran H., Durrington P.N. (2008). Hypercholesterolaemia and its management. BMJ.

[53] Petkova Z., Teneva O., Antova G., Angelova-Romova M., Gecheva G., Dimitrova-Dyulgerova I. (2023). Chemical composition, lipid-soluble bioactive compounds, and potential health benefits of the moss *Hypnum cupressiforme* Hedw. Plants.

